# Rethinking network analysis in ethnopharmacology: a multi-omics and AI roadmap to overcome conceptual and methodological biases

**DOI:** 10.3389/fphar.2026.1748478

**Published:** 2026-02-20

**Authors:** Xuewen Diao, Hao Zhang, Shiqi Wang, Zulong Wang, Qi Zhang

**Affiliations:** 1 The First Affiliated Hospital of Henan University of Chinese Medicine Department of Andrology, Zhengzhou, China; 2 The First Clinical Medical School, Henan University of Chinese Medicine, Zhengzhou, China; 3 Faculty of Chinese Medicine, Macau University of Science and Technology, Taipa, Macao SAR, China; 4 The First Affiliated Hospital of Henan University of Chinese Medicine Department, Zhengzhou, China

**Keywords:** artificial intelligence, ethnopharmacology, homogeneity, multi-omics, network analysis, network pharmacology

## Abstract

Network analysis (NA) is a widely used computational tool for exploring the complex systems of interactions in ethnopharmacology, aiming to predict potential targets and generate mechanistic hypotheses. However, the predictive validity and biological relevance of its outputs are constrained by a pervasive methodological bottleneck: the recurrent identification of a narrow set of molecules—such as quercetin—across disparate natural products and diseases. Through a systematic analysis of 1,038 network-based studies, we establish “homogeneity” as a coherent, multi-level pattern, from “Flavonoid Centrality” to a “Hub-Target Core” and restricted “Canonical Pathways,” transcending specific remedies or diseases. We conceptualize this as a self-reinforcing “convergent discovery pipeline,” in which initial database biases are amplified by context-insensitive analytical approaches. Empirical evidence shows that integrating contextual experimental or multi-omics data mitigates homogeneity. To break this cycle and align network analysis more closely with pharmacological best practices, we propose an integrated framework that shifts from database dependency to empirically driven data acquisition, leverages bias-aware artificial intelligence for curation and prioritization, and advances dynamic, context-specific network modeling. This framework provides a clear roadmap to disrupt methodological inertia and steer network-based research in ethnopharmacology toward a more robust, diverse, and pharmacologically and clinically relevant future.

## 1 Introduction

Ethnopharmacology explores the medicinal use of natural products across diverse cultural traditions, offering a rich resource for treating complex diseases. However, integrating such traditional knowledge into modern evidence-based pharmacology requires the identification of synergistic mechanisms underlying multi-component remedies—a task that poses significant challenges for conventional reductionist methods (Luqman et al., 2014; Li et al., 2017).

Network analysis (NA) has emerged as a widely adopted approach to address this complexity. Its systems-based framework is employed to map potential interactions among natural products, their constituents, biological targets, and pathways, thereby generating testable hypotheses regarding multi-target mechanisms (Lee et al., 2019). Consequently, network-based methodologies have become commonplace in contemporary ethnopharmacological research.

In its early development, this computational approach justifiably relied on accessible public databases and standardized workflows. This paradigm facilitated the translation of complex formulations into computable models and proliferated numerous hypotheses, playing a crucial role in establishing its utility. Yet as the discipline matures, the limitations of this standardized, predominantly database-dependent approach have grown apparent. A particularly widespread issue is the striking methodological homogeneity of core findings: across studies of disparate natural products and diseases, a narrow set of molecules—such as quercetin—is consistently identified as key active components (Zeng and Zhou, 2022). This pattern raises a critical question: does it reflect a genuine convergence on fundamental biological pathways, or does it expose a systemic bias toward pharmacologically questionable predictions ingrained within the computational discovery pipeline itself? If methodological in origin, such bias would represent a major impediment, undermining the exploratory potential of network analysis and distancing its outputs from the evidentiary standards required in ethnopharmacology.

To address this, we conducted a large-scale census of network analysis studies. Our objectives were to quantify the prevalence of output homogeneity and dissect its methodological origins, thereby proposing a framework to mitigate this bias and guide the field toward more pharmacologically relevant research.

## 2 Materials and methods

### 2.1 Search strategy and rationale

A systematic literature search was performed in the Web of Science Core Collection database on 13 December 2024, to identify original research articles published between 1 October 2023, and 30 June 2024. The search query TS = (“network pharmacology”) was used and restricted to Open Access original research articles to ensure transparency and verifiability. The retrieved dataset was finalized on the search date for subsequent analysis.

### 2.2 Inclusion and exclusion criteria

Studies were eligible if they applied a network analysis (often termed “network pharmacology”) framework to investigate mechanisms of natural products or botanical extracts. Exclusion criteria included: (1) studies not focused on natural products; (2) reviews, editorials, or conference abstracts; (3) duplicate publications; (4) unavailable full text; and (5) studies with incomplete or unclear data on key elements.

### 2.3 Data extraction and standardization

#### 2.3.1 Screening workflow and quality control

**FIGURE 1:**
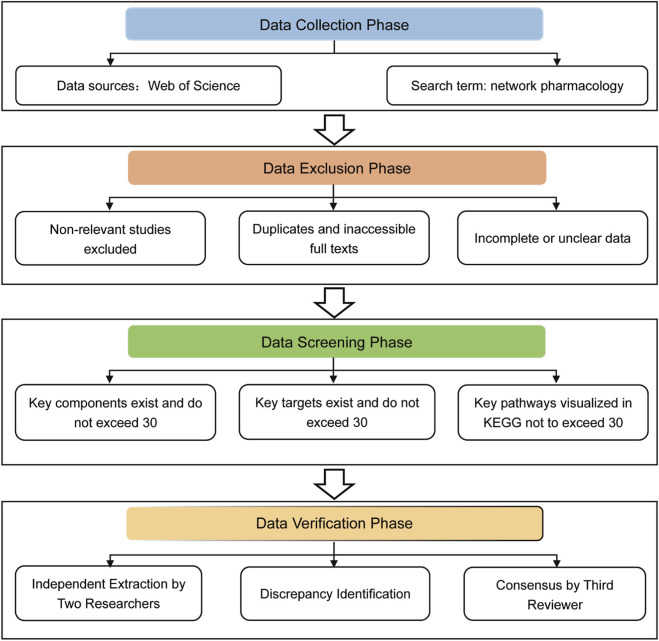
Article collection and screening workflow for homogeneity assessment.

The study selection process was conducted in accordance with the key elements of the PRISMA guidelines, particularly the systematic screening and selection procedures. Two researchers independently screened titles/abstracts and subsequently assessed full-text articles for eligibility. Data from included studies were extracted independently by the same two researchers. Discrepancies at any stage were resolved through consensus or consultation with a third reviewer. This workflow was designed to ensure reproducibility and minimize bias, as illustrated in Figure 1.

#### 2.3.2 Standardization of key findings identification

To address inconsistent terminology for core discoveries (e.g., “key targets,” “core targets”), we developed *a priori* glossaries that compiled a comprehensive set of relevant phrases for “key metabolites” and “key targets” (Supplementary File 1), identified through an initial review of the literature. Any list presented under these predefined headings was considered to represent the study’s key findings. This approach provided a reproducible rule set that captured the vast majority of reported key elements.

#### 2.3.3 Data extraction

Data were extracted separately for key metabolites, targets, and pathways. Extraction was contingent upon the study reporting no more than 30 items per category, applying the standardized glossary (Supplementary File 1). For pathways, we specifically extracted those visually presented as key findings in Kyoto Encyclopedia of Genes and Genomes enrichment analysis plots or explicitly listed in results. If a study presented multiple non-identical lists of key elements, the smaller, more refined set was extracted to capture the authors’ prioritized findings.

#### 2.3.4 Post-hoc nomenclature standardization of high-frequency elements

We focused this rigorous cleaning on the top 20 most frequent metabolites and targets. For these high-priority elements, we curated lists of their most common synonyms from authoritative databases (PubChem for metabolites, UniProt for targets) (see Supplementary File 1). For example, for a prevalent metabolite like quercetin, we unified its numerous high-frequency synonyms (e.g., “Sophoretin,” “Meletin”) under the standard name “Quercetin”. This targeted approach was implemented to mitigate the impact of nomenclature variation on the frequency counts of high-priority elements.

### 2.4 Statistical analysis

This study constitutes a systematic census of network-based studies from a defined period, thereby employing descriptive statistics (frequencies and percentages) to map the prevalence and distribution of “homogeneity” across the entire corpus.

To examine the impact of methodological choices on homogeneity, we pre-specified stratified analyses conducted separately for metabolites and targets:

For Metabolites: Studies were categorized as “Database-Dependent” (solely using computational predictions) or “Component Identification-Integrated” (incorporating experimental techniques such as mass spectrometry).

For Targets: Studies were classified as “Database-Dependent” (sourcing targets exclusively from prediction databases or protein-interaction networks) or “Omics-Integrated” (utilizing empirical data from transcriptomics, proteomics, or other multi-omics approaches).

We compared the prevalence (percentages) of top elements between these strata for metabolites and targets.

To evaluate the robustness of our homogeneity findings relative to the predefined threshold of 30 key elements per study, we conducted sensitivity analyses by recalculating prevalences using more restrictive thresholds of 20, 10, and 5 key elements.

## 3 Results

### 3.1 Literature screening

Our systematic search identified 1,301 publications. After eligibility screening, we included 1,038 articles for full-text review and data extraction (for the complete dataset, see Supplementary File 1). Cohorts for each analytical level were constructed as follows. For metabolite analysis, 465 articles provided usable data after exclusions (305 studies on specific compounds; 259 lacking relevant terminology; 8 exceeding the 30-metabolite threshold; 1 with incomplete data). For target analysis, 880 articles were included after exclusions (107 with no relevant target terms, including one study focused on a single pre-specified target; 49 exceeding the threshold; 2 with incomplete data). For pathway analysis, 917 articles qualified after exclusions (87 with no relevant terms; 30 exceeding the threshold; 4 with incomplete data).

### 3.2 Pervasive homogeneity across metabolites, targets, and pathways

Our large-scale analysis reveals homogeneity as a pervasive, systemic bias affecting all core levels of network analysis. As quantified in Table 1, a remarkably narrow set of elements dominates the reported findings. The recurrence is pronounced: quercetin—a flavonoid commonly flagged for its pan-assay interference potential—was reported as a key metabolite in nearly two-thirds of studies (63.2%). Similarly, AKT Serine/Threonine Kinase 1 (AKT1), a ubiquitous signaling hub protein, ranked first among targets, appearing in 48.8% of studies. Broad, well-annotated pathways such as PI3K-Akt and AGE-RAGE signaling were similarly prevalent, each highlighted in approximately half of all pathway analyses. This pattern of robust recurrence extends to subsequent ranks, with metabolites like beta-sitosterol, targets such as Interleukin-6 (IL6), and pathways including “Pathways in cancer” recurring in 25%–50% of the literature.

**TABLE 1:** Systemic homogeneity in network analysis: the most prevalent elements.

Category	Rank	Element/Target/Pathway	Prevalence (n/N)
Metabolite	1	Quercetin	63.2% (294/465)
	2	Kaempferol	47.9% (223/465)
	3	Luteolin	31.0% (144/465)
Target	1	AKT1	48.8% (429/880)
	2	TNF	39.1% (344/880)
	3	EGFR	34.8% (306/880)
Pathway	1	AGE-RAGE signaling pathway	54.9% (504/917)
	2	PI3K-Akt signaling pathway	54.7% (502/917)
	3	Lipid and atherosclerosis	50.2% (460/917)

Critically, this recurrence transcends specific research contexts. Whether a study focused on cancer, inflammation, or neurological disorders, the same limited set of “usual suspects” consistently emerged. This strong dissociation from specific disease pathobiology suggests the findings may be driven more by the methodological characteristics of the analytical pipeline than by the unique pathophysiology of each condition. Together, this quantitative evidence establishes a profound homogeneity that fundamentally constrains innovation in the field.

### 3.3 Sensitivity and stratified analyses

To assess the robustness of our findings, we conducted sensitivity analyses by varying the threshold for defining “key” elements. The results (Supplementary File 1) demonstrate that the dominance of top-ranked elements is highly stable across different stringency levels.

For metabolites, quercetin, kaempferol, and luteolin occupied the top three ranks under all thresholds (Top-30, -20, -10, and -5). Notably, quercetin remained the most frequent key metabolite even in the most restrictive analysis (Top-5), appearing in 62.8% (162/258) of studies. Furthermore, flavonoids consistently constituted 70%–80% of the top 20 most frequent metabolites, regardless of the threshold.

A similarly stable pattern was observed for targets. AKT1 retained its first-place ranking under all thresholds, identified as a key target in 35.6% (93/261) of studies even when analysis was restricted to the top 5 targets. A core group comprising Tumor Necrosis Factor (TNF), IL6, and Epidermal Growth Factor Receptor (EGFR) consistently appeared within the top six ranks across all thresholds, and the composition of the top 20 targets remained largely unchanged.

For pathways, the AGE-RAGE signaling pathway ranked first under the Top-30 threshold, while the PI3K-Akt signaling pathway was most prevalent under the more stringent Top-20 and Top-10 thresholds. Across all thresholds, these two pathways consistently occupied the top two ranks. The top pathways were predominantly large, generic signaling pathways (e.g., PI3K-Akt, AGE-RAGE, TNF) and broad disease-centric collections (e.g., Pathways in cancer).

Stratified analysis based on methodological approach revealed a significant mitigating effect of empirical data integration. As detailed in Table 2, the prevalence of the most homogenized elements was markedly reduced in studies that integrated experimental metabolite identification or multi-omics target data, compared to those relying solely on database-derived predictions (see Supplementary File 1 for complete ranked lists by strata).

**TABLE 2:** Comparative analysis of homogeneity: database-dependent vs. experimentally-validated or omics-integrated studies.

Category	Ranking	Database-dependent studies (Element, Prevalence)	Experiment/Omics-integrated studies (Element, Prevalence)
Metabolites	1	Quercetin (71.9%, 236/328)	Quercetin (42.3%, 58/137)
	2	Kaempferol (54.3%, 178/328)	Kaempferol (32.8%, 45/137)
	3	Beta-sitosterol (36.6%, 120/328)	Luteolin (21.2%, 29/137)
	4	Luteolin (35.1%, 115/328)	Naringenin (14.6%, 20/137)
	5	Stigmasterol (17.4%, 57/328)	Isorhamnetin (14.6%, 20/137)
Targets	1	AKT1 (54.9%, 398/725)	IL6 (24.2%, 37/153)
	2	TNF (42.6%, 309/725)	PTGS2 (24.2%, 37/153)
	3	EGFR (37.2%, 270/725)	EGFR (23.5%, 36/153)
	4	IL6 (35.3%, 256/725)	TNF (22.9%, 35/153)
	5	ESR1 (32.4%, 235/725)	ESR1 (21.6%, 33/153)

## 4 Discussion

### 4.1 The systemic pattern of homogeneity: from isolated finding to census

Our census of 1,038 NA studies reveals a striking and systemic convergence in reported findings across all levels of analysis—a pattern we term output homogeneity. This is not a sporadic occurrence but a coherent, multi-level phenomenon: studies investigating vastly different natural products and diseases consistently prioritize a narrow set of metabolites, targets, and pathways. This convergence suggests that the prevailing computational pipeline systematically channels outputs toward a restricted, and potentially misleading, biological narrative, largely independent of the specific research question.

This pattern manifests at every analytical level. At the metabolite level, “Flavonoid Centrality” is evident: quercetin was a key component in 63.2% of studies. Sensitivity analyses confirmed this bias—across all thresholds for defining “key” elements (Top 30, 20, 10, 5), flavonoids consistently comprised 70%–80% of the most frequent metabolites. This chemical dominance extends to targets, forming a “Hub-Target Core.” Within this core, proteins such as AKT1 (prevalent in 48.8% of studies) retain top ranking even under stringent criteria. Ultimately, pathway outputs converge into a restricted “Canonical Pathway” profile, dominated by a small, stable set of signaling pathways—most notably PI3K-Akt and AGE-RAGE—regardless of the analytical threshold applied.

The tight interconnection across these levels demonstrates that the current paradigm is exceptionally effective at generating a specific, predictable type of result. However, the critical question raised by our data is not merely about repetition, but about validity: when the same narrow narrative emerges from inquiries into disparate diseases, it challenges the pharmacological relevance of these computational findings. This convergence may indicate a systematic bias towards computationally convenient, but biologically or therapeutically questionable, predictions. Therefore, the pressing task is to move beyond documenting this pattern and to diagnostically uncover the methodological roots that generate such convergent—and potentially misleading—outputs.

### 4.2 Deconstructing the homogeneity: a three-tiered bias framework

The pervasive homogeneity documented in Section 4.1 is not random but the predictable product of a convergent discovery pipeline. This pipeline can be deconstructed into three sequential tiers where methodological choices systematically introduce and amplify bias: Input-Level Bias (originating from foundational data and selection criteria), Model-Level Bias (embedded in analytical algorithms), and Output-Level Convergence (the self-reinforcing cycle that perpetuates findings). The following sections detail how biases at each tier channel diverse research questions toward a homogenized narrative.

#### 4.2.1 Tier 1: input-level bias–the homogenized foundation

The homogeneity in NA findings originates from systemic biases embedded within foundational resources. This initial bias occurs across two interdependent domains: the chemical space of metabolites and the biological space of targets. Notably, these issues persist despite established NA guidelines advocating for high-quality data and comprehensive pharmacokinetic evaluation (Wang Z. Y. et al., 2022). These biases do not merely narrow the field of view; they systematically seed the analytical pipeline with candidates prone to being false positives or pharmacologically irrelevant predictions, a risk magnified when dealing with complex mixtures.

##### 4.2.1.1 Structural biases in foundational databases

Foundational databases (e.g., TCMSP for compounds; DisGeNET for disease associations) provide the standardized data essential for NA. However, as developing resources, they exhibit structural biases that constrain the discovery pipeline from its inception.

A primary limitation is compound databases' reliance on *in vitro* phytochemical profiles. These fail to capture critical *in vivo* pharmacokinetic processes—such as bioavailability, metabolic transformation, and tissue distribution—creating a “chemical–reality gap” (Dou et al., 2020; Chen et al., 2018). Critically, these profiles are predominantly qualitative, lacking data on constituent abundance. This absence of quantitative context creates a systemic bias: chemically stable compounds frequently reported across many plant species (e.g., common flavonoids like quercetin) become over-represented as “key” database entries, regardless of their pharmacologically relevant abundance *in vivo*.

This inherent database bias is powerfully amplified by common screening practices. Although guidelines advocate comprehensive Absorption, Distribution, Metabolism, and Excretion (ADME) evaluation, assessment is often reduced to applying two generic thresholds: Oral Bioavailability (OB) ≥ 30% and Drug-Likeness (DL) ≥ 0.18 (Guan et al., 2023; Peng et al., 2022; Anqi and Shijun, 2023). This simplified filter excludes potentially valuable agents that do not fit this profile—such as ginsenosides, which have low oral bioavailability but active metabolites (Seong et al., 2021)—while preferentially selecting flavonoids like kaempferol and quercetin that naturally satisfy these criteria. Together, database limitations and this algorithmic filtering establish the “Flavonoid Centrality” observed in our analysis.

Consequently, many studies risk prioritizing “chemical ghosts”—database-abundant compounds lacking evidence of meaningful systemic exposure. For example, quercetin is frequently identified as a key metabolite yet is often absent from the experimentally verified blood-absorbed constituents of the corresponding formula (Li et al., 2022). This mismatch indicates that mechanistic hypotheses anchored solely in database entries may be built on pharmacologically irrelevant compounds.

A parallel issue arises in disease-target databases. Designed for comprehensive coverage, they often list thousands of genes associated with a condition (Bao et al., 2022; Wang T. et al., 2022; Zhai et al., 2023). While inclusive, this practice inflates disease modules with broadly expressed “star” genes, diluting causal specificity. When these over-inclusive target sets intersect with pre-filtered compound-target networks, they preferentially retain ubiquitous, well-annotated proteins, further reinforcing a homogenized biological narrative.

Thus, the very resources meant to provide comprehensive starting points can, in practice, pre-constrain both the chemical and biological spaces of inquiry toward a narrow, overlapping set of high-frequency elements.

##### 4.2.1.2 The engine of bias: high-throughput screening and its computational echo

The prominence of flavonoids extends beyond database artifacts to the experimental root of the underlying interaction data: High-Throughput Screening (HTS). HTS is a powerful tool but is intrinsically prone to identifying compounds that act as pan-assay interference substances (PAINS), which produce false-positive signals across diverse assay formats due to non-specific mechanisms like chemical reactivity, aggregation, or fluorescence quenching (Yang et al., 2020; Bolz et al., 2021; Magalhães et al., 2021). These compounds are often reported as frequent hitters (FHCs), but their “activity” is an artifact of assay interference rather than specific target engagement.

Flavonoids are archetypal PAINS. Our analysis provides concrete evidence that the field’s computational data foundation is contaminated with such interferents: the top-ranking flavonoids in our census (e.g., quercetin, kaempferol) are canonical examples of PAINS frequently flagged in medicinal chemistry literature (Bisson et al., 2016). This means the apparent “broad bioactivity” of these compounds, which underpins many *in silico* predictions, is chemically misleading.

This experimental bias at the source creates a cascading effect. First, HTS platforms historically utilize limited target libraries enriched for well-studied, highly connected “star” proteins (e.g., AKT1, IL6) (Macarron et al., 2011; Malo et al., 2006) [, pre-homogenizing the biological target space. Second, and critically, computational prediction tools and the databases they populate (e.g., SwissTargetPrediction) are trained on or derived from these same biased HTS datasets. Consequently, they learn to echo the biases: they predict that PAINS will interact promiscuously with many targets, while consistently prioritizing the same limited set of star targets. This creates a self-reinforcing computational echo chamber that mathematically guarantees the “Hub-Target Core” observed in our findings.

A profound limitation of the current pipeline is that widely used, curated databases for ADME prediction and target identification fail to adequately filter or even flag these known PAINS. For instance, SwissADME—a commonly cited resource—has not seen a major update since 2019 and operates as a predictive aggregator rather than a critically curated repository (Daina et al., 2017). Its outputs and those of similar tools are linked in a network of mutually reinforcing predictions where false positives are not systematically assessed, and information (including PAINS-related artifacts) appearing in one database is likely to propagate uncritically to others. The core assumption in many such tools—that structural similarity implies similar activity at similar sites—represents the “biggest caveat of *in silico* design” when applied to promiscuous chemotypes like polyphenols (Zhong et al., 2022; Geppert et al., 2010). It leads to “blanket speculation” rather than specific, testable pharmacological hypotheses, as these tools cannot distinguish between a genuine, specific interaction and the non-specific interference inherent to PAINS.

In summary, the input bias originates from a contaminated experimental source (HTS/PAINS), which is then encoded, amplified, and legitimized by the computational tools and databases that form the backbone of standard network analysis workflows. This systemic failure to address fundamental pharmacological confounders at the data level is the primary reason why the pipeline generates stable, homogenized, and pharmacologically suspect predictions.

#### 4.2.2 Tier 2: model-level bias–the context-indifferent amplifier

If biased inputs provide homogenized and contaminated ingredients, the prevailing modeling paradigm acts as the recipe that systematically converts these flaws into a seemingly coherent, yet pharmacologically misleading, narrative. This paradigm is fundamentally “context-indifferent.” It not only overlooks the dynamic and tissue-specific nature of biological systems but, more critically, lacks the discriminatory power to separate genuine pharmacological signals from the noise of false positives and non-specific interactions seeded at the input level.

##### 4.2.2.1 The static network scaffold and the “hub” fallacy of degree centrality

The standard modeling approach employs a static and reductionist architecture. The common practice of building discrete “herb-component-target” networks compartmentalizes what is an integrative pharmacological reality (Chevereau and Bollenbach, 2015; Leigh et al., 2022), creating a static snapshot incapable of reflecting dynamics across tissues or disease stages (Wang et al., 2020; Zhang et al., 2022). This homogeneous analytical scaffold is the perfect stage upon which input biases can play out predictably across diverse research questions.

Operating on this flawed scaffold, the near-exclusive reliance on degree centrality to identify key nodes exemplifies a context-blind approach with severe pharmacological consequences. This metric treats all predicted interactions as equal, utterly disregarding critical dimensions such as binding affinity, functional effect (activation vs. inhibition), and tissue-specific expression (Adjei, 2006; Mei and Yang, 2018; Yao et al., 2018). Thus, it mathematically equates the promiscuous, non-specific connectivity of a PAINS compound (like quercetin) or a ubiquitous cellular hub protein (like AKT1) with high therapeutic relevance. In doing so, the method institutionalizes the misinterpretation of noise (false-positive interactions) as signal (key therapeutic targets).

Consequently, nodes already overrepresented in foundational data—promiscuous compounds like quercetin and ubiquitous “hub” targets like AKT1 (AKT Serine/Threonine Kinase 1)—are mechanically prioritized. Our stratified analysis confirms this artifact. Among database-dependent studies, AKT1 was a key target in 54.9% of cases (398/725). In contrast, its prevalence dropped to 19.6% (30/153) in studies integrating omics data. This dramatic contrast demonstrates that the dominance of such hubs is not a reflection of their unique therapeutic importance, but a direct artifact of a model that mistakes broad, non-specific network connectivity for pharmacological significance.

##### 4.2.2.2 The inflationary bias of over-representation analysis

At the pathway level, homogeneity is entrenched and legitimized by the routine use of over-representation analysis (ORA). ORA possesses an inherent size bias, favoring larger pathways because their greater gene count increases the likelihood of achieving statistical significance (Dong et al., 2016; Fang et al., 2012). Empirical studies show that an improperly defined background gene set can inflate the perceived significance of a pathway by up to 50-fold (Cao et al., 2025), meaning a pathway may be deemed “significant” primarily due to its size rather than its true contextual or causal relevance to the disease (Wieder et al., 2021).

Compounding this issue, the standard practice of ranking pathways solely by p-value erroneously equates statistical significance with biological or therapeutic importance—a profound fallacy that further severs the link between computational output and pharmacological reality (Lee, 2016; Maitland et al., 2007). Consequently, ORA acts as a powerful homogenizing and de-contextualizing filter. It consistently elevates large, generic signaling pathways (e.g., PI3K-Akt) and broad disease categories (e.g., Pathways in cancer) to prominence, not because they are mechanistically specific to the natural product or disease under study, but because they are statistically robust to the noise and bias present in the input target lists.

In summary, the “context-indifferent” paradigm—from its static network scaffold to its pharmacology-agnostic algorithms—forms the critical amplification pillar of the convergent pipeline. It does not merely combine biased inputs; it mathematically and rhetorically transforms them, giving a veneer of analytical rigor to what are often predictable, pharmacologically dubious, and homogenized outputs. It is the stage where the “promiscuous interactor” is crowned the “key therapeutic target.

#### 4.2.3 Tier 3: output-level convergence and the self-reinforcing cycle

The sequential action of input- and model-level biases leads to a convergent output: the recurrent identification of a narrow set of compounds, targets, and pathways. While this convergence intersects with some established biology [e.g., flavonoids modulating broad signaling pathways (Mansuri et al., 2014; Li et al., 2020)], the core issue is its systematic over-amplification by a pipeline that is highly prone to generating predictable results. A fundamental challenge is that such computational approaches will produce a set of “key” elements regardless of the specific query, which can lead to over-interpretation if not critically contextualized.

**FIGURE 2:**
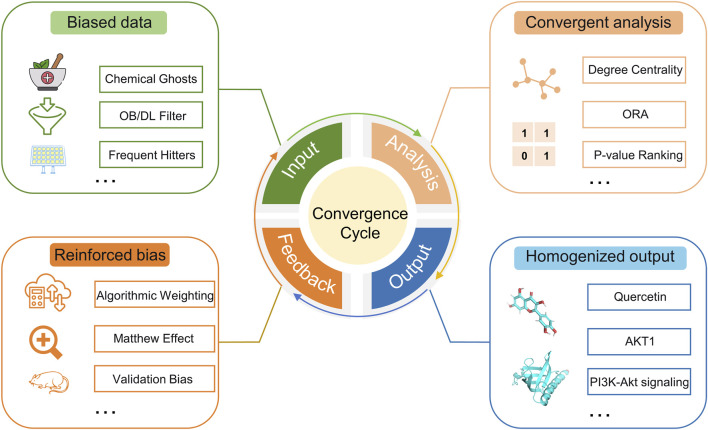
Concept map of homogenization cycle.

This convergent pipeline operates within a broader research ecosystem that inadvertently reinforces it, forming a self-reinforcing cycle (Figure 2). The cycle is propelled by three interdependent feedback mechanisms that can gradually solidify preliminary computational findings into perceived biological truths, often without the rigorous pharmacological scrutiny they require:

##### 4.2.3.1 Computational feedback

The Reinforcement of Predictive Patterns. Homogenized elements gain increased weight in database algorithms and predictive tools due to their frequent appearance in literature (Daina et al., 2019; Stelzer et al., 2016). This creates a feedback loop that can steer future analyses toward the same narrow candidate pool, making these patterns increasingly robust computationally, but not necessarily more valid pharmacologically.

##### 4.2.3.2 Sociological feedback

The Visibility of Familiar Narratives. Familiar, homogenized results tend to attract more citations and attention [the Matthew Effect (Jackson, 1988; Bol et al., 2018)], which can lead to their canonization in secondary literature. This visibility, while understandable, can inadvertently overshadow less obvious but potentially important novel insights, and may reduce the incentive to critically evaluate the underlying pharmacological evidence for each specific case.

##### 4.2.3.3 Resource allocation feedback

The Focus on “Validating” Popular Targets. Experimental resources often flow toward popular, computationally-derived targets to generate confirmatory data (Smaldino and McElreath, 2016; Edwards and Roy, 2017). However, a significant risk arises when this validation phase itself lacks rigor—for example, through the use of pharmacologically implausible *in vivo* doses or underpowered docking studies (Che and Zhang, 2025). This can create an illusion of robust, multi-methodological support for homogenized findings, while truly innovative and context-specific hypotheses struggle to attract the resources needed for their equally rigorous testing.

The cycle of biased data → context-insensitive analysis → homogenized output → uncritical reinforcement risks creating a growing gap between computational output and the standards of evidence-based pharmacology. It can lead to an “exploration bottleneck,” where research efforts are funneled toward re-examining a narrow set of canonical mechanisms. Consequently, the field’s knowledge base may become disproportionately populated with findings that are computationally convenient and self-reinforcing, rather than uniquely informative or therapeutically salient for specific herbal preparations.

Therefore, breaking this cycle of homogeneity requires reforms that must extend beyond the analytical methodology (input, model, output) to establish a non-negotiable prerequisite at the study’s inception: rigorous scrutiny of the preparation itself. The current pipeline’s stability in generating homogenized and pharmacologically suspect predictions is fundamentally enabled by its allowance for analyses built upon “chemical ghosts”—entities detached from the actual pharmacological evidence of the evaluated preparation. Future network analyses must, therefore, originate from a critical appraisal and explicit reporting of the preparation’s pharmacological evidence base, encompassing its chemical definition, evidence for *in vivo* exposure of key constituents, and dose-relevance. Only by anchoring computational analyses to such an examined reality can the subsequent technical framework reforms—from data acquisition to dynamic modeling—effectively steer the field toward genuinely innovative and reliable hypothesis generation.

### 4.3 Toward a pharmacologically grounded future: a framework for credible hypothesis generation in network analysis

**FIGURE 3:**
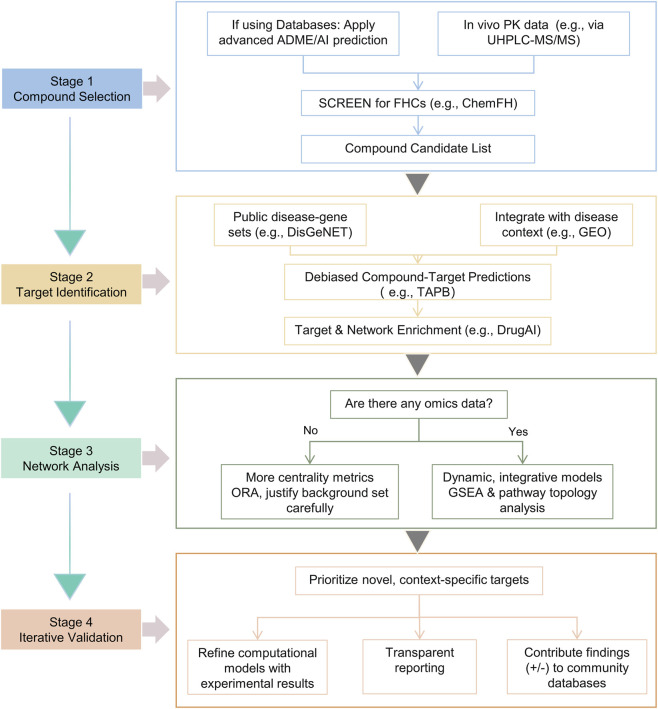
A strategic, phased roadmap for mitigating methodological homogeneity in network analysis. The flowchart illustrates the four-stage workflow (Compound Selection, Target Identification, Network Analysis, Iterative Validation) and the key decision points within each stage. It explicitly distinguishes between the minimum viable (left/default paths) and ideal (right/empirical paths) implementation levels discussed in the text. Specific actions and tools are highlighted at each step (e.g., screening for FHCs with ChemFH, integrating disease-context from GEO/TCGA, selecting analytical methods based on available data context).

The pervasive convergence on unreliable predictions necessitates a fundamental evolution in the application of network analysis. To align it with the pharmacological best practices required in ethnopharmacology, we propose an integrated framework. This framework is built upon a foundational principle that precedes and informs all technical improvements: network analysis must be grounded in a critical appraisal of the pharmacological evidence for the specific preparation under study. Only with this premise secured can the framework then shift its focus from generating any statistically plausible output to producing pharmacologically credible and context-specific hypotheses. It emphasizes that computational tools must be embedded within a rigorous, evidence-driven research cycle where their primary value lies in prioritizing targets for subsequent experimental validation, not in declaring mechanistic insights. The following sections detail this framework, culminating in a phased roadmap for implementation (Section 4.3.4; Figure 3).

#### 4.3.1 Paradigm shift: from database dependency to empirically driven data acquisition

Our analysis demonstrates that shifting from database-derived predictions to empirically grounded data is a decisive strategy for reducing the reliance on false leads and pharmacologically questionable predictions. This shift, as shown in Table 2, concomitantly markedly reduces the prevalence of homogenized elements at the field level. For instance, quercetin’s identification as a key metabolite dropped from 71.9% to 42.3%, and AKT1’s prevalence as a key target fell from 54.9% to 19.6%.

To pinpoint the mechanism behind this improvement, we conducted a controlled re-analysis of a study on Taohong Siwu Decoction (Li et al., 2025). We created a parallel workflow that differed only in two inputs: (1) using a TCMSP-derived constituent list rather than the experimentally verified blood-absorbed compounds, and (2) selecting disease-relevant targets from generic databases instead of intersecting predictions with experimental metabolomic data.

The divergence was stark and systematic. The database-driven path prioritized “chemical ghosts”—specifically quercetin, luteolin, kaempferol, baicalein, and β-sitosterol—none of which were detected in the original plasma metabolome. In contrast, the original study identified *in vivo*-confirmed constituents like ferulic acid and vanillic acid as key bioactives. Crucially, these “ghost” compounds are archetypal homogenized elements. They exhibited extraordinarily high numbers of predicted targets (223, 125, 119, 118, and 109, respectively), with an average of 139 predicted interactions—far exceeding the background average of ∼32 per compound in our dataset. These compounds exemplify how database-centric approaches can systematically prioritize molecules that are not only homogenized but are also high-risk candidates for generating false-positive pharmacological narratives due to their absence *in vivo* and/or PAINS-like properties. Consequently, the inferred core target sets diverged fundamentally. The original, empirically-anchored analysis identified a target set (e.g., JUN, PTGS2, BCL2, ESR1, PPARG). The database path yielded a distinct set dominated by ubiquitous, high-degree proteins (TP53, HSP90AA1, AKT1, JUN, EP300). Notably, AKT1—the most homogenized target in our census—was not linked to any experimentally confirmed constituents. It entered the network solely through interactions predicted for the “ghost” flavonoids and other database-specific compounds (e.g., beta-carotene).

It is important to note that this case study is illustrative rather than comprehensive. Its purpose is not to provide definitive proof for a specific formula’s mechanism, but to concretely demonstrate how methodological choices at the input stage (database vs. empirical data) can systematically divert the analytical trajectory toward or away from homogenized elements. This case demonstrates that the shift to empirical data is not merely a diversification tactic, but a critical strategy to bypass the false leads generated by database artifacts and PAINS-driven predictions, thereby grounding hypotheses in biologically observable events. Together, the field-level statistics and this mechanistic case study provide compelling, multi-level evidence for the proposed shift.

This evidence underscores the necessity of a paradigm shift in data acquisition, based on two core principles:

Prioritize *In Vivo* Exposure for Ingredient Screening. “*In vivo* detectability” should be a primary criterion. Techniques like Ultra High Performance Liquid Chromatography-Tandem Mass Spectrometry (UHPLC-MS/MS) should be used to identify key constituents, thereby circumventing database-driven “chemical ghosts” and ensuring that hypotheses are built on compounds with actual pharmacological opportunity (Zhang et al., 2021; Wei et al., 2022).

Anchor Target Identification in Pathophysiological Context. Target prioritization must integrate multi-omics data (e.g., transcriptomics, metabolomics) that reflect the specific disease context. This anchors target hypotheses in disease biology, helping to discriminate genuine therapeutic associations from the false-positive “hub” targets that arise from topology-driven analysis. (Chung and Kang, 2019).

To systematically implement these principles, we propose establishing a data evidence hierarchy within network analysis guidelines. This hierarchy would formally recognize the superior value of context-specific empirical data:

Highest Priority (Tier 1): Data from *in vivo* pharmacokinetic studies of the specific formula or herb, identifying prototype compounds and metabolites actually present in systemic circulation or target tissues.

Secondary Consideration (Tier 2): Constituent lists from generic phytochemical databases (e.g., TCMSP). When used, methodological limitations must be explicitly acknowledged—for example: “The compound list was sourced from database X, which is based on *in vitro* phytochemical profiles; the *in vivo* relevance and relative abundance of these compounds for the studied formula were not confirmed in this work.”

Such a framework encourages transparency and mandates that researchers justify their data sources while actively seeking higher-quality evidence. Studies should also report specific analytical methods (e.g., UHPLC-MS/MS) and any steps taken to cross-verify database entries, such as comparison with literature on absorbed components.

Building on this foundation, we advocate for a complementary, community-driven strategy of “co-development and enhancement.” This involves establishing shared data standards and incentivizing researchers to contribute validated empirical data to public platforms. The strategy focuses on two synergistic actions: (1) co-developing dedicated repositories for high-quality *in vivo* pharmacokinetic and multi-omics data, and (2) enhancing existing general databases by integrating this empirical evidence with higher confidence weighting, using clear annotations such as “*In Vivo* Verified” or “Tissue-Specific Expression.”

By adopting a clear evidence hierarchy and fostering open data collaboration, the research community can progressively counterbalance the overrepresentation of “high-frequency components” in predictive databases. This will steer network analysis toward a more dynamic, evidence-informed, and self-correcting knowledge foundation.

#### 4.3.2 Leveraging AI for bias-aware data curation and prioritization

To disrupt the cycle of unreliable predictions, we advocate for a strategic, pharmacologically-informed application of artificial intelligence. AI should be leveraged not as a black-box oracle, but as a tool to explicitly address the identified sources of pharmacological irrelevance: screening for interference compounds, correcting for target bias, and modeling functional outcomes. This shifts AI’s role from a passive predictor to an active curator for pharmacologically plausible hypotheses, thereby increasing the likelihood that computational outputs will lead to meaningful experimental validation.

At the input stage, AI can directly counter “Flavonoid Centrality” by identifying and accounting for chemically promiscuous or assay-interfering compounds. Integrated platforms such as ChemFH exemplify this approach. ChemFH is an online tool that screens for frequent false positives—including colloidal aggregators, fluorescent compounds, and promiscuous molecules—using deep learning models trained on large datasets of known interferents (Shi et al., 2024). Incorporating such tools into virtual screening workflows allows researchers to algorithmically down-weight high-risk candidates, reducing the systematic over-prioritization of nonspecific bioactive compounds from the outset. Furthermore, AI-powered pharmacokinetic predictors (e.g., BioTransformer) facilitate a move beyond simplistic OB/DL filters by offering nuanced predictions of *in vivo* absorption and metabolic fate (Wishart et al., 2022; Fang et al., 2023). This helps prioritize a more diverse and pharmacologically realistic set of candidate constituents. To institutionalize this step, future methodological guidelines should encourage—or even mandate—the reporting of such screenings, requiring transparency on how potential FHCs were identified and handled.

Beyond input filtering, advanced AI models are essential for correcting the predictive biases that reinforce the “Hub-Target Core.” The field must shift from predicting binary interactions to modeling context-specific and functionally annotated relationships. Frameworks such as the Target-bias Aware Prediction Benchmark (TAPB) explicitly address prior bias toward highly studied targets in drug–target interaction models, providing a blueprint for debiased prediction (Lin et al., 2025). Concurrently, models like DrugAI predict not merely binding but also the direction of pharmacological effect (activation/inhibition), adding a crucial layer of functional context for target prioritization (Zhang et al., 2023). This allows researchers to evaluate targets based on their putative mechanistic role within a disease-relevant pathway, rather than on topological promiscuity alone. Furthermore, capturing the critical influence of structural heterogeneity on bioactivity—where slight chemical modifications can dramatically alter a compound’s effect—requires moving beyond simplistic molecular representations (Stumpfe and Bajorath, 2012). AI-driven models excel at this by learning from nuanced features such as 3D conformations and pharmacophores, offering a significant advantage over traditional linear notations (e.g., SMILES) in distinguishing between chemically similar yet pharmacologically distinct compounds, such as different flavonoids (Gu et al., 2025; An et al., 2022).

Finally, AI serves as the cornerstone for building the next-generation of equitable, evidence-weighted knowledge foundations—a critical step for long-term bias mitigation. Platforms like TCMBank and BATMAN-TCM exemplify this direction (Lv et al., 2023; Kong et al., 2024). TCMBank intelligently integrates and manually curates compound–target–disease relationships from vast literature and multiple databases, creating a consolidated resource that reduces the fragmentation and inherent biases of single-source repositories. BATMAN-TCM further incorporates multi-omics data to enable context-aware screening. On the technological front, automated literature-mining systems (leveraging models like BioBERT) can continuously scan and cross-verify findings, flagging inconsistencies for expert review and gradually cleansing the knowledge ecosystem of self-perpetuating errors (Peng et al., 2020; Lee et al., 2020; Hwang et al., 2021). To support transparency, the use of such integrated knowledge bases should be encouraged, and studies should report complete target lists to enable critical appraisal and meta-analysis.

In summary, by consciously directing AI’s capabilities toward input bias detection, debiased and functional prediction, and robust knowledge integration, we can transform it into a powerful engine for generating pharmacologically credible hypotheses. This structured, AI-informed strategy is essential for systematically dismantling the cycle of unreliable predictions and rebuilding a discovery pipeline whose outputs are both novel and primed for rigorous experimental confirmation.

#### 4.3.3 Beyond static snapshots: empirical evidence and prospects for multi-dimensional dynamic networks

To generate hypotheses that are both mechanistically insightful and readily testable, network analysis must advance from static snapshots toward a dynamic and context-aware modeling paradigm. This evolution addresses the core pharmacological limitation of current methods and would concomitantly reduce methodological homogeneity. This evolution is supported by emerging research and would benefit from a corresponding shift in methodological guidelines.

Strategically integrating multi-omics data is central to this transition. Moving beyond linear associations requires computational frameworks capable of fusing heterogeneous data types, such as transcriptomics, proteomics, and metabolomics. Two principal strategies show promise: mixed integration, which transforms diverse datasets into a unified analytical format, and hierarchical integration, which organizes data by biological scale (e.g., molecular, cellular, tissue) to respect inherent regulatory relationships (Liu et al., 2020; Mariette and Villa-Vialaneix, 2018; Picard et al., 2021). AI techniques—including multi-view learning and graph neural networks—are key to implementing these strategies, enabling models that reflect systemic complexity and can simulate drug action across varied contexts.

Pioneering frameworks illustrate this potential. SETComp uses deep learning to identify cell-specific mechanisms, moving beyond static hub-target assumptions (Wang et al., 2025). Herb-CMap integrates perturbed transcriptomic data with network propagation to reveal critical targets overlooked by conventional analyses (Wang et al., 2024). Platforms like POINT demonstrate how combining multi-omics networks with knowledge graphs can counteract biases inherent in single-layer approaches (He et al., 2025).

A critical observation, however, is that even advanced frameworks like Herb-CMap can still output homogenized elements—such as quercetin, AKT1, and PI3K-Akt signaling—when their input data are drawn from the narrow chemical-biological space common in the field. This critical observation underscores that algorithmic sophistication alone cannot rescue pharmacologically questionable inputs. It reinforces the imperative for the paradigm shift outlined in Section 4.3.1: enriching the pipeline with context-rich, dynamic data is a prerequisite for generating novel and reliable hypotheses, not just a technical enhancement.

To support this evolution, we propose several extensions to future methodological guidelines, grounded in the issues identified in our analysis:

Encourage temporally and conditionally resolved data. Where feasible, guidelines should promote the use of time-series or dose-response omics data. This would shift the focus from static comparisons toward modeling pharmacodynamic trajectories and contextual responses, thereby providing testable predictions about kinetic profiles and dose-dependencies that are central to pharmacological evaluation.

Advance beyond topology-only metrics. While useful, simple metrics like degree centrality often mistake broad connectivity for therapeutic relevance. Guidelines could emphasize context-aware measures—such as betweenness centrality (identifying network bottlenecks), eigenvector centrality (weighting influential nodes), or network propagation seeded with disease-specific data—to better prioritize targets with pathological specificity, and away from targets prioritized solely by their propensity to be false-positive hubs.

Promoting robust pathway interpretation. To counter the size bias of ORA, guidelines could encourage methods like Gene Set Enrichment Analysis (GSEA) or pathway topology analysis. When ORA is used, transparent reporting of background gene sets and discussion of potential biases should be encouraged to prevent the misinterpretation of statistical artifacts as biological insight.

Fostering iterative and transparent validation. Guidelines should reinforce the importance of context-aware experimental validation of computational predictions. Supporting an iterative cycle where results refine models, and reporting complete target lists and key parameters, would enhance reproducibility and enable community evaluation.

In summary, advancing toward dynamic, context-aware models is not a purely computational exercise. Its paramount value lies in generating hypotheses that are temporally resolved, dose-dependent, and mechanistically nuanced—attributes that make them inherently more testable and pharmacologically relevant than the static, homogenized outputs of current practices. This evolution from static to dynamic modeling is, therefore, not an optional upgrade but an essential re-engineering of the hypothesis generation process, designed to close the gap between computational prediction and actionable, pharmacologically rigorous experimental design.

#### 4.3.4 A phased roadmap for methodological diversification

To translate our conceptual framework into practice and enhance accessibility for groups with varying resources, we propose a phased roadmap structured around the core workflow of network analysis. This roadmap is designed to accommodate a spectrum of implementation levels—from a minimum viable approach that focuses on critical, resource-efficient interventions, to an ideal implementation that fully realizes the framework’s potential. Both pathways are designed with a common, overriding objective: to incrementally increase the pharmacological credibility and testability of the hypotheses generated. This means that even resource-constrained “minimum viable” steps should be explicitly chosen to mitigate the most egregious sources of false positives and biological irrelevance, paving a principled (if incremental) path toward more reliable discovery (Figure 3).

#### 4.3.5 Stage 1: refining compound selection

##### 4.3.5.1 Minimum viable approach

When dedicated *in vivo* studies are not feasible, rigorously screen database-derived compound lists for FHCs using accessible tools (e.g., ChemFH). Move beyond the simplistic OB/DL ≥ 30/0.18 filter by employing more comprehensive, freely available ADME predictors (e.g., SwissADME) or AI-based tools (e.g., BioTransformer). Transparently report all sources, filters, and screening outcomes. Even when *in vivo* data is unavailable, these steps represent a minimum commitment to pharmacological realism by actively filtering out the most egregious sources of false positives (PAINS) and moving beyond simplistic, misleading filters.

##### 4.3.5.2 Ideal implementation

Prioritize compounds with direct evidence of *in vivo* exposure, identified through dedicated pharmacokinetic studies (e.g., UHPLC-MS/MS of plasma samples). Supplement this with FHC screening. The long-term objective for this stage is to cultivate community-shared repositories of high-quality *in vivo* pharmacokinetic data and to develop next-generation, bias-aware prediction tools specifically designed for the unique chemical space of natural products.

#### 4.3.6 Stage 2: contextualizing target identification

The goal of this stage is to transform a generic list of predicted interactions into a prioritized shortlist of targets that justify experimental investment. To counter the “Hub-Target Core,” target lists must be explicitly linked to the disease context, ensuring that limited experimental resources are directed toward testing targets with a higher prior probability of specific, mechanistically meaningful engagement.

##### 4.3.6.1 Minimum viable approach

When disease-relevant omics data are available in public repositories, intersect predicted compound-target interactions with disease-context gene sets (e.g., GEO for transcriptomics, TCGA for cancer genomics). Crucially, employ or prioritize prediction models/algorithms that explicitly account for or correct prior target bias (e.g., through negative sampling, regularization, or using benchmark frameworks designed to assess debiasing). Clear documentation of target sources and the rationale for addressing bias is critical.

##### 4.3.6.2 Ideal implementation

Anchor target identification in primary, disease-specific multi-omics data (e.g., transcriptomics/proteomics from relevant models). Integrate these empirical signatures with advanced, debiased prediction models or custom analytical pipelines to maximize specificity. Employing models that predict functional effects (e.g., activation/inhibition) provides a deeper, mechanistically informed layer of context. The future direction involves the routine integration of multi-dimensional data on drug action (e.g., cellular phenotyping, spatial omics) to build a fully resolved, mechanistic understanding of compound-target relationships.

#### 4.3.7 Stage 3: advancing network and pathway analysis

##### 4.3.7.1 Minimum viable approach

Supplement or replace sole reliance on degree centrality with other accessible centrality measures (e.g., betweenness, eigenvector) to capture different aspects of node importance. If using ORA, carefully justify the choice of background gene set and explicitly acknowledge its inherent size bias. While still limited, these steps move beyond the methods most prone to identifying generic, false-positive hubs (degree centrality) and inflated pathways (ORA), thereby yielding a slightly more refined set of candidates for downstream validation.

##### 4.3.7.2 Ideal implementation

Move beyond basic centrality and ORA by employing more sophisticated, context-aware algorithms. For network analysis, this includes methods such as network propagation algorithms or the use of integrated platforms (e.g., POINT) that can incorporate prior biological knowledge or multi-omics data to refine node prioritization. For pathway analysis, prefer Gene Set Enrichment Analysis (GSEA) or pathway topology analysis over ORA when gene-level data are available, as they better account for gene correlations and pathway structure. The ultimate goal is to develop and adopt predictive, multi-dimensional dynamic network models capable of simulating pharmacological interventions across biological contexts, thereby transitioning from static association to causal, systems-level prediction.

#### 4.3.8 Stage 4: closing the loop with iterative validation

It is non-negotiable and defines the ultimate purpose of the entire framework. It represents the essential transition from computational speculation to pharmacological evidence. The credibility of any network analysis study is contingent upon its commitment to this validation loop. This stage transforms computational “predictions” into pharmacological “evidence.” Its output is not merely a publication, but empirical data that retrospectively validates the computational workflow and prospectively enriches the community knowledge base.

##### 4.3.8.1 Minimum viable approach

Prioritize a subset of high-confidence, non-canonical targets for experimental testing in disease-relevant models. Report both positive and negative results transparently.

##### 4.3.8.2 Ideal implementation

Establish a full iterative cycle where validation results are used to refine the computational model. Contribute all validated findings—with clear annotations on the experimental context—to community databases to enrich the shared knowledge base.

This roadmap provides a practical path for implementing the paradigm shift advocated throughout this article. Its long-term vision is the establishment of a rigorous, self-correcting research discipline where network analysis reliably serves the generation of high-value hypotheses, and where iterative validation closes the loop, ensuring computational tools evolve in tandem with pharmacological evidence.

## 5 Challenges and limitations

### 5.1 Limitations of this study

Our study has several limitations. First, the analysis was restricted to open-access articles in the Web of Science Core Collection, which, while ensuring reproducibility, may not capture the entirety of NA research, particularly studies in non-English languages. Second, our identification of “key findings” relied on a standardized glossary, which might have missed elements presented only in graphical abstracts or unstructured narratives, though we judge this impact on the overall macroscopic trend to be minimal. We also clarify that our goal is to highlight a systemic methodological bias, not to disregard the value of individual studies that have advanced our understanding of specific formulae.

### 5.2 Potential challenges in implementing the proposed framework

Translating our proposed framework—which aims to ground network analysis in pharmacological verifiability—into practice faces significant hurdles. Technically, obtaining comprehensive *in vivo* pharmacokinetic data for complex ethnopharmacology mixtures remains costly and methodologically challenging (Xu et al., 2021). The biologically meaningful integration of multi-omics data and the construction/validation of dynamic network models require novel algorithms and substantial computational resources (Subramanian et al., 2020).

Critically, the application of AI within this domain encounters unique obstacles. First, leveraging Natural Language Processing for mining TCM literature is complicated by specialized terminology, ambiguous compound names, and the need to reconcile classical descriptions with modern biomedical concepts, posing distinct challenges for automated knowledge extraction. Second, while “bias-aware AI” is essential, there is an inherent risk that poorly designed models may learn and amplify existing biases rather than correcting them (Obermeyer et al., 2019). This underscores the necessity for Explainable AI (XAI) techniques. In the context of generating testable hypotheses, XAI is not merely a “nice-to-have” but a prerequisite, as it allows researchers to understand the rationale behind a prioritized target, thereby informing smarter experimental design and validation strategies (Rudin, 2019).

On the methodological front, while we critique “context-blind” metrics, the field has yet to converge on a new generation of robust, “context-aware” alternatives. Key questions remain: What network measures can best capture tissue specificity or dynamic criticality?

Furthermore, implementing this data- and AI-intensive framework introduces broader systemic challenges. The push for open data sharing must be meticulously balanced with robust safeguards for patient privacy, particularly for sensitive clinical and multi-omics data (Sung, 2023). Equally important is addressing algorithmic fairness; models trained on non-representative data risk perpetuating health disparities, necessitating diverse datasets and bias audits (Yang et al., 2024).

Addressing this interconnected set of challenges is central to the mission of re-engineering network analysis as a disciplined component of ethnopharmacological research. Only by doing so can it move beyond generating self-reinforcing artifacts and fulfill its potential as a rigorous, hypothesis-generating tool that reliably informs—rather than misdirects—experimental and clinical translation.

## 6 Conclusion

In conclusion, our analysis identifies methodological homogeneity as a symptom of a deeper crisis in the application of network analysis within ethnopharmacology: a disconnect from pharmacological rigor and a proliferation of low-evidence predictions. To unlock its potential, we propose a framework pivoting from database dependency to empirical, AI-enhanced, and dynamic modeling, explicitly subordinated to the evidentiary standards of the field. This shift is designed not to revalidate canonical findings, but to reposition network analysis as a powerful, yet humble, tool for generating testable hypotheses, thereby enabling the discovery of novel, context-specific therapeutic mechanisms and fostering a more robust future for ethnopharmacology research.

## Data Availability

The original contributions presented in the study are included in the article/Supplementary Material, further inquiries can be directed to the corresponding authors.
