# Supplementary material for: Rethinking network analysis in ethnopharmacology: a multi-omics and AI roadmap to overcome conceptual and methodological biases
Source: Front Pharmacol. 2026 Feb 20;17:1748478. doi: 10.3389/fphar.2026.1748478 (PMC12962898; doi:10.3389/fphar.2026.1748478)
Supplement: Supplementary file 1 [file Table1.docx]

**Supplementary Table 1 terminology for key metabolites**

| Category | Terms |
| --- | --- |
| Components | principal bioactive components, key active components, important components, main Active Components, most crucial components, representative active components, main functional components, core components, major chemical components,effective chemical components |
| Ingredients | core active ingredients,key ingredients, important roles, main active ingredients, top active ingredients, extremely active ingredients, primary active ingredients, |
| Compounds | key compounds, most important potential active compounds, most active compounds, important compound, the compounds of high value, essential role, principal active compounds, most relevant compounds, most important compounds, best compound, main bioactive compounds, top active identified compounds, significant functional compounds, essential compound, therapeutic compounds, crucial multifunctional bioactive compounds |
| Constituents | key constituents, main active constituents, core constituents, selected phytochemical constituents, |
| Others | critical active substances, important roles, have critical parts in the treatment of KOA,crucial roles, top contributors, core role, particularly promising candidate, top contents, core candidate |

Supplementary Table 2 terminology glossary key targets

| Category | Terms |
| --- | --- |
| Targets | core common targets, core targets, potential therapeutic targets, potential drug targets, key therapeutic targets, central therapeutic targets, main targets, major targets, primary targets, promising targets, most likely XXX-related targets, high-priority targets,hub targets |
| Proteins | core proteins, key proteins, main target proteins, closely related proteins, hub proteins |
| Genes | key genes, core genes, crucial genes, functional genes, top genes, top identified genes, most important genes, hub genes, characteristic genes |
| Others | core roles, important roles, crucial roles, significant players, essential role, most important nodes |

Supplementary Table 3 common synonyms key metabolites

| Standard name | Common Synonyms |
| --- | --- |
| quercetin | quercetin, 117-39-5,Quercetine, Quercetol, Quercitin, Meletin, Sophoretin, 3,3',4',5,7-Pentahydroxyflavone , 2-(3,4-Dihydroxyphenyl)-3,5,7-trihydroxy-4H-chromen-4-one, CHEBI:16243,CHEMBL50,DB04216,Quertine, Quertin, Kvercetin, Quer, QUE, 3,5,7,3',4'-Pentahydroxyflavone, Xanthaurine |
| Kaempferol | 520-18-3, Kaempherol, Kempferol, Kampferol, Robigenin, Trifolitin, Pelargidenolon, Nimbecetin, 3,4',5,7-Tetrahydroxyflavone, 3,5,7-Trihydroxy-2-(4-hydroxyphenyl)-4H-chromen-4-one, CHEBI:28499, UNII-731P2LE49E, NSC-407289, C.I. 75640, 5,7,4'-Trihydroxyflavono |
| Luteolin | 491-70-3, Luteoline, Digitoflavone, Luteolol, Flacitran, 3',4',5,7-Tetrahydroxyflavone, 2-(3,4-Dihydroxyphenyl)-5,7-dihydroxy-4H-chromen-4-one, CHEBI:15864, UNII-KUX1ZNC9J2, C.I. 75590, Cyanidenon 1470, 5,7,3',4'-Tetrahydroxyflavone, NSC-267432, SR-01000779333, MFCD00017309 |
| Beta-Sitosterol | 83-46-5, Sitosterol, B-Sitosterol, beta-Sitosterin, 22,23-Dihydrostigmasterol, Quebrachol, Cinchol, (3beta)-Stigmast-5-en-3-ol, 24alpha-Ethylcholesterol, CHEBI:27693, UNII-S347WMO6M4, NSC-8096, alpha-Dihydrofucosterol, Stigmast-5-en-3beta-ol, Cupreol, Harzol |
| Isorhamnetin | 480-19-3, Isorhamnetol, 3-Methylquercetin, Quercetin 3'-methyl ether, 3'-O-Methylquercetin, 3'-Methoxyquercetin, 3,4',5,7-Tetrahydroxy-3'-methoxyflavone, 3,5,7-Trihydroxy-2-(4-hydroxy-3-methoxyphenyl)-4H-chromen-4-one, CHEBI:6052, UNII-07X3IB4R4Z, C.I. 75680, NSC-267432, 3,5,7,4'-Tetrahydroxy-3'-methoxyflavone, MFCD00017310, IRH |
| Stigmasterol | 83-48-7, Stigmasterin, beta-Stigmasterol, Stigmasta-5,22-dien-3beta-ol, (24S)-5,22-Stigmastadien-3beta-ol, (3beta,22E)-Stigmasta-5,22-dien-3-ol, CHEBI:28824, UNII-99WUK5D0Y8, NSC-8095, Stigmasta-5,22E-dien-3beta-ol, 5,22-Stigmastadien-3beta-ol, Serposterol, Wulzen anti-stiffness factor, MFCD00003630 |
| Naringenin | 480-41-1, (S)-Naringenin, (2S)-Naringenin, Salipurol, Naringenine, (-)-Naringenin, 5,7,4'-Trihydroxyflavanone, (2S)-5,7-dihydroxy-2-(4-hydroxyphenyl)-2,3-dihydrochromen-4-one, CHEBI:17846, UNII-HN5425SBF2, NSC-11855, Naringetol, salipurpol, pelargidanon, MFCD00870553, 4',5,7-Trihydroxyflavanone, Naringenin |
| Baicalein | 491-67-8, Noroxylin, 5,6,7-Trihydroxyflavone, 5,6,7-trihydroxy-2-phenyl-4H-chromen-4-one, CHEBI:2979, UNII-49QAH60606, NSC-661431, BaiKalein, Biacalein, MFCD00017459, 5,7-Trihydroxyflavone, Sho-saiko-to, SR-01000597499 |
| Formononetin | 485-72-3, Biochanin B, Formononetol, Neochanin, 7-Hydroxy-4'-methoxyisoflavone, 7-hydroxy-3-(4-methoxyphenyl)chromen-4-one, CHEBI:18088, UNII-295DQC67BJ, NSC-93360, 4'-O-methyldaidzein, Flavosil, Myconate, MFCD00016948, Daidzein 4-methyl ether |
| Wogonin | 632-85-9, Vogonin, 5,7-Dihydroxy-8-methoxyflavone, 5,7-dihydroxy-8-methoxy-2-phenyl-4H-chromen-4-one, Norwogonin 8-methyl ether, CHEBI:10043, UNII-POK93PO28W, NSC-717845, MFCD00017736, 5,7-Dihydroxy-8-methoxy-2-phenyl-4H-1-benzopyran-4-one, NSC-717845, SR-05000002216 |
| Apigenin | 520-36-5, Apigenine, Apigenol, Versulin, Spigenin, 4',5,7-Trihydroxyflavone, 5,7-Dihydroxy-2-(4-hydroxyphenyl)-4H-chromen-4-one, Pelargidenon 1449, C.I. Natural Yellow 1, CHEBI:18388, UNII-7V515PI7F6, NSC-83244, 5,7,4'-Trihydroxyflavone, MFCD00006831, Chamomile |
| Caffeic acid | 331-39-5, 3,4-Dihydroxycinnamic acid, trans-Caffeic acid, (E)-3-(3,4-Dihydroxyphenyl)acrylic acid, 3,4-Dihydroxybenzeneacrylic acid, Caffeate, 3-(3,4-Dihydroxyphenyl)-2-propenoic acid, CHEBI:16433, UNII-U2S3A33KVM, NSC-57197, MFCD00004392, Cinnamic acid, 3,4-dihydroxy-, (E)-3-(3,4-Dihydroxyphenyl)prop-2-enoic acid, 3,4-Dihydroxycinnamate, NSC-623438 |
| Rutin | 153-18-4, Rutoside, Quercetin 3-rutinoside, Quercetin-3-O-rutinoside, Phytomelin, Sophorin, Birutan, Myrticolorin, Quercetin 3-O-beta-D-rutinoside, Quercetin-3-rutinoside, C.I. 75730, CHEBI:28527, UNII-5G06TVY3R7, NSC-9220, 3-Rutinosyl quercetin, Violaquercetrin, Eldrin, Venoruton, MFCD00006830 |
| Ferulic acid | 1135-24-6, trans-Ferulic acid, (E)-Ferulic acid, 4-Hydroxy-3-methoxycinnamic acid, 3-(4-Hydroxy-3-methoxyphenyl)acrylic acid, Coniferic acid, (E)-3-(4-Hydroxy-3-methoxyphenyl)acrylic acid, Fumalic acid, CHEBI:17620, UNII-AVM951ZWST, NSC-2821, Ferulate, 3-methoxy-4-hydroxycinnamic acid, MFCD00004400, NSC-51986 |
| Hederagenin | 465-99-6, Caulosapogenin, Hederagenol, Astrantiagenin E, Hederagenic acid, (3beta)-3,23-dihydroxyolean-12-en-28-oic acid, CHEBI:69579, UNII-RQF57J8212, NSC-24954, cyclocaric acid A, Herderagenin, Hederagenine, MFCD00017385, (3beta,4alpha)-3,23-Dihydroxyolean-12-en-28-oic acid |
| Nobiletin | 478-01-3, 5,6,7,8,3',4'-Hexamethoxyflavone, 2-(3,4-Dimethoxyphenyl)-5,6,7,8-tetramethoxy-4H-1-benzopyran-4-one, 3',4',5,6,7,8-Hexamethoxyflavone, Hexamethoxyflavone, NSC-76751, CHEBI:7602, UNII-D65ILJ7WLY, NSC-618903, MFCD03273560, 2-(3,4-dimethoxyphenyl)-5,6,7,8-tetramethoxychromen-4-one |
| Baicalin | 21967-41-9, Baicalein 7-O-glucuronide, Baicalein 7-glucuronide, 5,6-dihydroxy-4-oxo-2-phenyl-4H-chromen-7-yl beta-D-glucopyranosiduronic acid, Baicalein 7-O-beta-D-glucuronide, CHEBI:2981, UNII-347Q89U4M5, MFCD00134418, Baicalein-7-D-glucuronide, 5,6,7-trihydroxyflavone-7-O-beta-D-glucuronate, 7-D-Glucuronic acid-5,6-dihydroxyflavone |
| Acacetin | 480-44-4, Linarigenin, 5,7-Dihydroxy-4'-methoxyflavone, 5,7-dihydroxy-2-(4-methoxyphenyl)chromen-4-one, 4'-Methoxyapigenin, Apigenin 4'-methyl ether, Acacetine, CHEBI:15335, UNII-KWI7J0A2CC, NSC-76061, Buddleoflavonol, Linarisenin, 4'-O-Methylapigenin, MFCD00016936 |
| Calycosin | 20575-57-9, 3'-hydroxyformononetin, 7,3'-dihydroxy-4'-methoxyisoflavone, 7-hydroxy-3-(3-hydroxy-4-methoxyphenyl)chromen-4-one, 3-Hydroxyformononetin, CHEBI:17793, UNII-09N3E8P7TA, MFCD00210598, 3',7-dihydroxy-4'-methoxyisoflavone, 7-hydroxy-3-(3-hydroxy-4-methoxyphenyl)-4H-chromen-4-one |
| berberine | berberine, 2086-83-1, 2353, Berberine chloride, Umbellatine, Berberin, CHEBI:16118, CHEMBL12089, C20H18NO4+ |

Supplementary Table 4 common synonyms key targets

| Standard name | Common Synonyms |
| --- | --- |
| AKT1 | P31749, RAC, Protein kinase B (PKB),Protein kinase B alpha (PKB alpha), Proto-oncogene c-Akt, RAC-PK-alpha, |
| TNF | P01375, Tumor necrosis factor, Cachectin, TNF-alpha, TNF-a, TNFSF2 |
| EGFR | P00533, Proto-oncogene c-ErbB-1, Receptor tyrosine-protein kinase erbB-1, ERBB, ERBB1, HER1 |
| IL6 | P05231, IL-6 , P05231, IFNB2, B-cell stimulatory factor 2, BSF-2, Hybridoma growth factor, Interferon beta-2 |
| ESR1 | P03372, ER, ER-alpha, Estradiol receptor, Nuclear receptor subfamily 3 group A member 1, ESR, NR3A1 |
| TP53 | P04637, p53, Cellular tumor antigen p53, Antigen NY-CO-13, Phosphoprotein p53, Tumor suppressor p53 |
| PTGS2 | P35354, Prostaglandin G/H synthase 2, EC:1.14.99.1, Cyclooxygenase-2, COX-2, PHS II, Prostaglandin H2 synthase 2, PGH synthase 2, PGHS-2, Prostaglandin-endoperoxide synthase 2 |
| CASP3 | P42574, Caspase-3, CASP-3, EC:3.4.22.56, Apopain, Cysteine protease CPP32, CPP-32, Protein Yama, SREBP cleavage activity 1, SCA-1 |
| JUN | P05412, Transcription factor Jun, c-Jun, Proto-oncogene c-Jun, Transcription factor AP-1 subunit Jun, V-jun avian sarcoma virus 17 oncogene homolog, p39 |
| STAT3 | P40763, STAT-3, Signal transducer and activator of transcription 3, Acute-phase response factor, APRF |
| VEGFA | P15692, VEGF-A, Vascular endothelial growth factor A, VEGF, Vascular permeability factor, VPF |
| MMP9 | P14780, Matrix metalloproteinase-9, MMP-9, EC:3.4.24.35, Gelatinase B, GELB, 92 kDa gelatinase, 92 kDa type IV collagenase |
| SRC | P12931, Proto-oncogene tyrosine-protein kinase Src, EC:2.7.10.2, Proto-oncogene c-Src, pp60c-src, p60-Src, c-Src |
| IL1B | P01584, Interleukin-1 beta, IL-1β, IL-1 beta, Catabolin |
| MAPK1 | P28482, Mitogen-activated protein kinase 1, MAP kinase 1, Extracellular signal-regulated kinase 2, ERK2, ERK-2,PRKM1, PRKM2, EC:2.7.11.24, p42-MAPK, MAP kinase isoform p42 |
| HSP90AA1 | P07900, Heat shock protein HSP 90-alpha, EC:3.6.4.10, Heat shock 86 kDa, HSP 86, HSP86, Heat shock protein family C member 1, Lipopolysaccharide-associated protein 2, LAP-2, LPS-associated protein 2, Renal carcinoma antigen NY-REN-38, HSPC1, HSPCA |
| PPARG | P37231, Peroxisome proliferator-activated receptor gamma, PPAR-gamma, Nuclear receptor subfamily 1 group C member 3,NR1C3, PPARγ |
| MAPK3 | P27361, PRKM3, Extracellular signal-regulated kinase 1, ERK1, ERK-1, p44-ERK1,Mitogen-activated protein kinase 3, MAP kinase 3, MAPK 3, p44-MAPK, MAP kinase isoform p44, EC:2.7.11.24 |
| RELA | Q04206, p65,Transcription factor p65,Nuclear factor NF-kappa-B p65 subunit, NFKB3, Nuclear factor of kappa light polypeptide gene enhancer in B-cells 3 |
| MAPK14 | Q16539, p38α / p38 alpha, Mitogen-activated protein kinase 14, AP kinase p38 alpha, Stress-activated protein kinase 2a (SAPK2a), EC:2.7.11.24, Cytokine suppressive anti-inflammatory drug-binding protein (CSAID), CSBP (CSAID-binding protein), MAX-interacting protein 2 (MXI2) |

**Supplementary Table 6. Sensitivity Analysis of Metabolite Homogeneity** under **Different Top-N Thresholds**

| Rank | Top-30 Threshold (n=465) | Top-20 Threshold (n=452) | Top-10 Threshold (n=401) | Top-5 Threshold (258) |
| --- | --- | --- | --- | --- |
| 1 | quercetin (294) | quercetin (284) | quercetin (255) | quercetin (162) |
| 2 | Kaempferol (223) | Kaempferol (213) | Kaempferol (183) | Kaempferol (105) |
| 3 | luteolin (144) | luteolin (141) | luteolin (124) | luteolin (80) |
| 4 | Beta-sitosterol (132) | Beta-sitosterol (128) | Beta-sitosterol (111) | Beta-sitosterol (62) |
| 5 | Isorhamnetin (74) | Isorhamnetin (70) | Isorhamnetin (53) | Naringenin (23) |
| 6 | Stigmasterol (64) | Stigmasterol (59) | Naringenin (45) | Stigmasterol (20) |
| 7 | Naringenin (62) | Naringenin (58) | Stigmasterol (45) | baicalein (19) |
| 8 | baicalein (49) | baicalein (48) | baicalein (42) | Isorhamnetin (19) |
| 9 | Formononetin (42) | Formononetin (38) | Wogonin (32) | Wogonin (16) |
| 10 | Wogonin (36) | Wogonin (35) | Formononetin (30) | nobiletin (10) |
| 11 | Apigenin (20) | Apigenin (19) | Apigenin (15) | apigenin (9) |
| 12 | Caffeic acid (19) | Caffeic acid (16) | nobiletin (14) | Formononetin (6) |
| 13 | Rutin (18) | Rutin (16) | hederagenin (13) | Rutin (6) |
| 14 | Ferulic acid (16) | nobiletin (15) | Tanshinone iia (11) | baicalin (5) |
| 15 | hederagenin (16) | Acacetin (14) | berberine (10) | berberine (5) |
| 16 | nobiletin (16) | Baicalin (14) | Calycosin (10) | hederagenin (5) |
| 17 | Baicalin (15) | hederagenin (14) | Rutin (10) | oleic acid (5) |
| 18 | Acacetin (14) | Ferulic acid (13) | Acacetin (9) | tanshinone IIA (5) |
| 19 | Calycosin (14) | berberine (12) | baicalin (9) | Acacetin (4) |
| 20 | berberine (13) | Calycosin (12) | Licochalcone A (9) | aloe-emodin (4) |

**Supplementary Table 7. Sensitivity Analysis of Targets Homogeneity** under **Different Top-N Thresholds**

| Rank | Top-30 Threshold (n=880) | Top-20 Threshold (n=825) | Top-10 Threshold (n=662) | Top-5 Threshold (261) |
| --- | --- | --- | --- | --- |
| 1 | AKT1 (429) | AKT1 (390) | AKT1 (294) | AKT1 (93) |
| 2 | TNF (344) | TNF (320) | TNF (241) | TNF (78) |
| 3 | EGFR (306) | IL6 (271) | IL6 (201) | IL6 (57) |
| 4 | IL6 (293) | EGFR (265) | EGFR (173) | IL1B (39) |
| 5 | ESR1 (268) | ESR1 (228) | TP53 (159) | TP53 (39) |
| 6 | TP53 (236) | TP53 (216) | ESR1 (152) | EGFR (37) |
| 7 | PTGS2 (226) | CASP3 (201) | PTGS2 (138) | VEGFA (37) |
| 8 | CASP3 (224) | PTGS2 (201) | IL1B (135) | PTGS2 (36) |
| 9 | JUN (216) | JUN (192) | CASP3 (134) | ESR1 (28) |
| 10 | STAT3 (215) | VEGFA (191) | VEGFA (133) | SRC (26) |
| 11 | VEGFA (213) | STAT3 (188) | STAT3 (132) | CASP3 (25) |
| 12 | MMP9 (211) | IL1B (187) | SRC (130) | MMP9 (24) |
| 13 | SRC (207) | MMP9 (182) | JUN (117) | STAT3 (24) |
| 14 | IL1B (202) | SRC (180) | MMP9 (114) | JUN (21) |
| 15 | MAPK1 (197) | MAPK1 (162) | MAPK1 (103) | HSP90AA1 (17) |
| 16 | HSP90AA1 (170) | HSP90AA1 (139) | HSP90AA1 (91) | MAPK1 (17) |
| 17 | PPARG (137) | PPARG (116) | PPARG (72) | PPARG (14) |
| 18 | MAPK3 (122) | MAPK3 (107) | MAPK3 (66) | ALB (11) |
| 19 | RELA (105) | RELA (95) | RELA (63) | RELA (12) |
| 20 | MAPK14 (102) | HIF1A (92) | HIF1A (49) | AR (10) |

**Supplementary Table 8. Sensitivity Analysis of Pathway Homogeneity** under **Different Top-N Thresholds**

| Rank | Top-30 Threshold (n=917) | Top-20 Threshold (n=762) | Top-10 Threshold (n=190) |
| --- | --- | --- | --- |
| 1 | AGE-RAGE signaling pathway (504) | PI3K-Akt signaling pathway (401) | PI3K-Akt signaling pathway (75) |
| 2 | PI3K-Akt signaling pathway (502) | AGE-RAGE signaling pathway (394) | AGE-RAGE signaling pathway (60) |
| 3 | Lipid and atherosclerosis (460) | Lipid and atherosclerosis (358) | Lipid and atherosclerosis (60) |
| 4 | Pathways in cancer (416) | Pathways in cancer (353) | TNF signaling pathway (60) |
| 5 | Proteoglycans in cancer (409) | Proteoglycans in cancer (313) | IL-17 signaling pathway (56) |
| 6 | TNF signaling pathway (399) | TNF signaling pathway (304) | Pathways in cancer (51) |
| 7 | Prostate cancer (382) | Prostate cancer (288) | Prostate cancer (41) |
| 8 | Hepatitis B (377) | Hepatitis B (285) | MAPK signaling pathway (40) |
| 9 | IL-17 signaling pathway (375) | IL-17 signaling pathway (285) | Proteoglycans in cancer (40) |
| 10 | HIF-1 signaling pathway (363) | HIF-1 signaling pathway (277) | Hepatitis B (38) |
| 11 | Fluid shear stress and atherosclerosis (352) | Fluid shear stress and atherosclerosis (257) | HIF-1 signaling pathway (38) |
| 12 | Kaposi sarcoma-associated herpesvirus infection (324) | Kaposi sarcoma-associated herpesvirus infection (247) | Apoptosis pathway (30) |
| 13 | Human cytomegalovirus infection (313) | Human cytomegalovirus infection (234) | Kaposi sarcoma-associated herpesvirus infection (30) |
| 14 | Endocrine resistance (306) | MAPK signaling pathway (230) | Endocrine resistance (29) |
| 15 | MAPK signaling pathway (296) | Endocrine resistance (220) | Fluid shear stress and atherosclerosis (28) |
| 16 | Chemical carcinogenesis - receptor activation (267) | EGFR tyrosine kinase inhibitor resistance (193) | Chemical carcinogenesis -- receptor activation (27) |
| 17 | EGFR tyrosine kinase inhibitor resistance (258) | Chemical carcinogenesis - receptor activation (191) | Human cytomegalovirus infection (27) |
| 18 | Apoptosis pathway (222) | Apoptosis pathway (158) | NF-kappa B signaling pathway (27) |
| 19 | Pancreatic cancer (214) | Pancreatic cancer (145) | Toll-like receptor signaling pathway (26) |
| 20 | Bladder cancer (199) | Bladder cancer (142) | EGFR tyrosine kinase inhibitor resistance (22) |

Supplementary Table 9. Stratified analysis of Pathway Homogeneity : Database-Dependent vs. Experiment-Integrated Studies.

| Ranking | Database-Dependent Studies (Element, Prevalence) | Experiment-Integrated Studies (Element, Prevalence) |
| --- | --- | --- |
| 1 | Quercetin (71.9%, 236/328) | Quercetin (42.3%, 58/137) |
| 2 | Kaempferol (54.3%, 178/328) | Kaempferol (32.8%, 45/137) |
| 3 | Beta-sitosterol (36.6%, 120/328) | Luteolin (21.2%, 29/137) |
| 4 | luteolin (35.1%, 115/328) | Isorhamnetin (14.6%, 20/137) |
| 5 | Stigmasterol (17.4%, 57/328) | Naringenin (14.6%, 20/137) |
| 6 | Isorhamnetin (16.5%, 54/328) | Formononetin (10.2%, 14/137) |
| 7 | Naringenin (12.8%, 42/328) | Beta-sitosterol (8.8%, 12/137) |
| 8 | baicalein (11.6%, 38/328) | Wogonin (8.8%, 12/137) |
| 9 | Formononetin (8.5%, 28/328) | Baicalein (8.0%, 11/137) |
| 10 | wogonin (7.3%, 24/328) | Chlorogenic acid (8.0%, 11/137) |
| 11 | hederagenin (4.6%, 15/328) | Caffeic acid (7.3%, 10/137) |
| 12 | Apigenin (3.4%, 11/328) | Apigenin (6.6%, 9/137) |
| 13 | baicalin (3.4%, 11/328) | Ferulic acid (6.6%, 9/137) |
| 14 | Rutin (3.4%, 11/328) | Calycosin (5.1%, 7/137) |
| 15 | Acacetin (3.0%, 10/328) | Hesperetin (5.1%, 7/137) |
| 16 | Tanshinone iia (3.0%, 10/328) | Nobiletin (5.1%, 7/137) |
| 17 | (+)-Catechin (2.7%, 9/328) | Rutin (5.1%, 7/137) |
| 18 | Caffeic acid (2.7%, 9/328) | Stigmasterol (5.1%, 7/137) |
| 19 | diosgenin (2.7%, 9/328) | Catechin (4.4%, 6/137) |
| 20 | Licochalcone a (2.7%, 9/328) | ellagic acid (4.4%, 6/137) |

Supplementary Table 10. Stratified analysis of Pathway Homogeneity : Database-Dependent vs. Omics-Integrated Studies.

| Ranking | Database-Dependent Studies (Element, Prevalence) | Omics-Integrated Studies (Element, Prevalence) |
| --- | --- | --- |
| 1 | AKT1 (54.9%, 398/725) | IL6 (24.2%, 37/153) |
| 2 | TNF (42.6%, 309/725) | PTGS2 (24.2%, 37/153) |
| 3 | EGFR (37.2%, 270/725) | EGFR (23.5%, 36/153) |
| 4 | IL6 (35.3%, 256/725) | TNF (22.9%, 35/153) |
| 5 | ESR1 (32.4%, 235/725) | ESR1 (21.6%, 33/153) |
| 6 | TP53 (28.6%, 207/725) | IL1B (21.6%, 33/153) |
| 7 | CASP3 (27.7%, 201/725) | AKT1 (19.6%, 30/153) |
| 8 | STAT3 (26.9%, 195/725) | JUN (19.6%, 30/153) |
| 9 | SRC (26.8%, 194/725) | MMP9 (19.6%, 30/153) |
| 10 | PTGS2 (26.1%, 189/725) | TP53 (18.3%, 28/153) |
| 11 | VEGFA (25.8%, 187/725) | VEGFA (17.0%, 26/153) |
| 12 | JUN (25.7%, 186/725) | CASP3 (15.0%, 23/153) |
| 13 | MAPK1 (25.5%, 185/725) | STAT3 (13.1%, 20/153) |
| 14 | MMP9 (25.0%, 181/725) | HSP90AA1 (12.4%, 19/153) |
| 15 | IL1B (23.3%, 169/725) | HIF1A (11.8%, 18/153) |
| 16 | HSP90AA1 (20.8%, 151/725) | FOS (9.2%, 14/153) |
| 17 | PPARG (17.9%, 130/725) | CCL2 (8.5%, 13/153) |
| 18 | MAPK3 (15.0%, 109/725) | MAPK3 (8.5%, 13/153) |
| 19 | RELA (13.8%, 100/725) | SRC (8.5%, 13/153) |
| 20 | MAPK14 (12.4%, 90/725) | TLR4 (8.5%, 13/153) |
